# Therapeutic effect and mechanism of electroacupuncture at Zusanli on plasticity of interstitial cells of Cajal: a study of rat ileum

**DOI:** 10.1186/1472-6882-14-186

**Published:** 2014-06-07

**Authors:** Mei-fang Peng, Kun Li, Chao Wang, Xiao-yan Zhu, Zheng Yang, Guo-hu Zhang, Pei-hong Wang, Yong-hua Wang, Li-jun Tang, Lin Zhang

**Affiliations:** 1 Basic Laboratory for PLA medical center, Chengdu General Hospital of Chengdu Military Area Command, Chengdu 610083, China; 2 Insititute of Biotechnology and Nuclear Technology, Sichuan Academy of Agricultural Sciences, Chengdu 610061, China; 3 Department of Preclinical Medicine, Chengdu Medical College, Chengdu 610500, China; 4 Center of General Surgery, Chengdu General Hospital of Chengdu Military Area Command, 270 Rongdu Rd.,Tianhui Town, Jinniu District, Chengdu 610083, China

**Keywords:** Electroacupuncture, Plasticity, DOG1, Inflammatory mediators, c-Kit signaling

## Abstract

**Background:**

Electroacupuncture (EA) is one of the techniques of acupuncture and is believed to be an effective alternative and complementary treatment in many disorders. The aims of this study were to investigate the effects and mechanisms of EA at acupoint Zusanli (ST36) on the plasticity of interstitial cells of Cajal (ICCs) in partial bowel obstruction.

**Methods:**

A Sprague Dawley rat model of partial bowel obstruction was established and EA was conducted at Zusanli (ST36) and Yinglingquan (SP9) in test and control groups, respectively. Experiments were performed to study the effects and mechanisms of EA at Zusanli on intestinal myoelectric activity, distribution and alteration of ICCs, expression of inflammatory mediators, and c-Kit expression.

**Results:**

*1*) EA at Zusanli somewhat improved slow wave amplitude and frequency in the partial obstruction rats. *2*) EA at Zusanli significantly stimulated the recovery of ICC networks and numbers. *3*) the pro-inflammatory mediator TNF-α and NO activity were significantly reduced after EA at Zusanli, However, no significant changes were observed in the anti-inflammatory mediator IL-10 activity. *4*) EA at Zusanli re-expressed c-Kit protein. However, EA at the control acupoint, SP9, significantly improved slow wave frequency and amplitude, but had no effect on ICC or inflammatory mediators.

**Conclusions:**

We concluded that EA at Zusanli might have a therapeutic effect on ICC plasticity, and that this effect might be mediated via a decrease in pro-inflammatory mediators and through the c-Kit signaling pathway, but that the relationship between EA at different acupoints and myoelectric activity needs further study.

## Background

Interstitial cells of Cajal (ICCs) play an important role in the regulation of gastrointestinal (GI) motility. In the GI tract, they act as pacemakers that generate and propagate slow waves, as mediators between the enteric nervous system and smooth muscle, and as stretch sensors [1,2]. Clinical studies have shown that the GI motility disorder of slow transit constipation (STC) is characterized by a reduction in ICC number and an impairment of the ICC cellular network [3-5]. The mechanism by which the ICC loss may be reversed in these patients remains a subject of interest. Clarification of the regulatory factors that control survival, disorder and proliferation of ICCs will promote the understanding and treatment of STC.

Previous studies have indicated that the interaction of the specific receptor tyrosine kinase c-Kit with its ligand stem cell factor (SCF) is essential for the development, differentiation, and functional maintenance of ICC in the intestine [6-9]. The c-Kit receptor is a well-established marker for ICC, and several human gastrointestinal motility disorders have been associated with depletion of Kit-positive ICC [10-12]. Inactivation of Kit with neutralizing antibodies or Kit inhibitors leads to defects in ICCs networks and the pacemaker activity involved in GI movement [6,13,14]. Re-expression of Kit protein may contribute to the recovery of ICC networks after they are disrupted by intestinal disorders [15,16]. Therefore, because the c-Kit/SCF signaling pathway is essential to the development and maintenance of ICC networks, if we could rescue ICC through reactivation of the c-Kit/SCF signaling pathway, we might find a new mode of therapy for STC patients.

GI inflammation has been linked to changes structure, number and function of ICC. The inflammatory cytokines, TNF-α and IL-4, can down-regulate the expression of c-Kit, and cause a reduction in the number of Kit-positive ICCs that is unrelated to apoptosis and is probably a trans-differentiation of the ICC to a type of α-smooth muscle action (α-SMA) positive cell with a smooth muscle cell phenotype [17]. Inflammatory mediators can also induce overexpression of inducible nitric oxide (NO) synthase (iNOS), increasing the concentration of NO and leading to defects in ICC networks [18-20]. Therefore, under the pro-inflammatory conditions in the tunica muscularis that are associated with intestinal obstruction, the release of bioactive substances, possibly from activated resident macrophages, may affect smooth muscle contractility. And, under these conditions, both the number and the function of neighboring ICC may be affected.

Electroacupuncture (EA) is one of the techniques of acupuncture within traditional Chinese medical practice, and is believed to be an effective alternative and complementary treatment in many disorders [21]. The Zusanli acupoint (ST36) is one of the most common acupoints used to treat GI disorders and has been reported to increase intestinal myoelectric activity and colonic motility and affect ICC networks in rats [22-24]. However, whether EA at Zusanli can cause recovery of the ICC phenotype or proliferation of ICC through inducing the release of bioactive mediators has not yet been investigated.

In the current study, the effect of EA treatment at Zusanli on the plasticity of ICCs was investigated. To gain insight into the underlying mechanism, we investigated whether EA at this acupoint affected the release of inflammatory mediators, and whether this treatment altered myenteric slow wave characteristics and ICC morphology. We also investigated how EA treatment at Zusanli could affect the plasticity of ICCs, perhaps through the c-Kit/SCF signaling pathway, or through an influence on inflammatory mediators.

## Methods

### Animal model of partial bowel obstruction

Twenty-five adult Sprague Dawley (SD) rats (180–250 g, male or female, aged 6–8 weeks) were purchased from the Animal Center of the Third Military Medical University (Chongqing, China) and used in our experiments. The animal model of partial bowel obstruction was produced according to the method published by Chang et al. [15]. Briefly, the rats were anesthetized with pentobarbital sodium (Nembutal 50 μg · g^−1^). After the absence of a hind-limb pinch-withdrawal reflex was verified, a loop of intestine was exposed through a midline laparotomy, and a medical latex clip (6 mm in length, 6 mm external diameter, 5 mm internal diameter) placed over a piece of intestine 30–50 mm oral to the ileocaecal sphincter. The clip size was designed to reduce the ability of a bolus of material to pass through the site, but to avoid obstruction of the resting diameter. Following placement of the clip, the clip was sutured with 5–0 silk suture thread, the intestine returned into the abdomen, and the abdomen sutured shut. A control group of sham-operated rats was also prepared. These animals underwent the same surgical preparation but had no clip installed. All animals including experimental animals, un-operated controls and sham-operated controls were cared for with regular feeding before and after surgery. The animal experiments were performed in accordance with our Hospital of Health Guide for the Care and Use of Laboratory Animals. The study was approved by the Institutional Ethics Committee of Chengdu General Hospital of Chengdu Military Area Command.

### Experimental groups and protocol overview

Animals were divided into five groups (n = 5 in each group): (1) normal (N group), (2) sham-operated (S group), (3) operated without EA treatment (O-N group), (4) operated and EA treatment at the Zusanli acupoint (O-EZ group), (5) operated and EA treatment at the SP9 (Yinglingquan) acupoint (O-EY group), the acupoint control group. In preliminary experiments, the abdomen was opened at Day 16 to confirm that the segment proximal to the clip was dilated 150-300% and that the model was therefore successful. After these preliminary experiments indicated that distension and hypertrophy due to partial bowel had developed by 15 days after clip placement, we used the following protocol: Day 1 – surgery to produce partial bowel obstruction or sham surgery; Days 31 – 37, daily EA in the O-EZ and O-EY groups; Day 38 – recording of slow waves, taking of blood samples, followed by euthanasia and removal of tissue samples.

### EA treatment

EA stimulation was provided at two acupoints: the experimental acupoint ST36 Zusanli (5 mm below head of fibula under the knee joint and 2 mm lateral to the anterior tubercle of the tibia) and a control acupoint SP9 Yinglingquan (in a depression anterior and inferior to the head of the fibula). SP9 belongs to the spleen meridian and Zusanli to the stomach meridian of Foot Taiyin. The stomach meridian and the spleen meridian are corresponding acupoints, the stomach meridian being on the outside and the spleen meridian on the inside of the lower leg. Therefore SP9 is in a corresponding position to Zuzanli. Also, having both acupoints in the same region made the experimental procedure easier to handle. Hwato needles (diameter, 0.2 mm; length, 7 mm; Suzhou Medical Appliance Factory, Suzhou, China) were inserted in a perpendicular manner into the acupoints to a depth of about 5 mm, and electric stimulation was provided through the needles by an EA treatment device (Great wall KWD-808, Hangzhou, China). Stimulation parameters were chosen according to the experience of the author in traditional Chinese medicine, which says that the muscles of the lower limb should be stimulated to be vibrated slightly. These stimulation parameters were disperse-dense waves of 5/20 Hz (28.5 ms/15 ms pulse duration) frequency and 2–4 mA current density. Stimulation intensity was adjusted to a level that elicited a slight muscle twitch at the acupuncture site and was limited to maximum of 3 mA in order to minimize animal discomfort. After preliminary experiments confirmed that 30 min EA daily for 7 days did not induce EA tolerance, rats in the O-EZ and O-EY groups received EA treatment daily for 30 min for one week.

### Intestinal myoelectric studies

The day after the last EA treatment (Day 38) all animals were anaesthetized and a laparotomy was performed. Myoelectric slow wave activity was recorded at the following three regions of the small intestine: 5 mm oral to the occlusion clip, 6 mm beneath the clip, and 50 mm aboral to the clip. Two silver electrodes were implanted parallel to each other in each region; one end of the electrode was inserted into the intestinal seromuscular layer, and the other end connected to the bioelectricity amplifier. Electric signals acquired at a sample frequency of 100 Hz, with a high-pass cutoff filter set at 20 Hz, scanning rate at 1.00 s/div and scanning time was 30 min, were then processed and stored in a standard computer. Data analysis was performed according to the method published by Tomita [25]. For signal processing, 1 min recording sessions were designated as a segment, and ten segments were sampled in a random manner in every animal. Several electrical parameters were analyzed: (1) slow wave amplitude, (2) frequency, (3) standard deviation and coefficient of variation.

### Immunofluorescence observation of ICC

ICCs were identified by immunolabeling for DOG1 as previously described [15,26-29]. DOG1 labels all classes of ICC and represents a highly specific marker for studying the distribution of ICC in mouse and human tissues. It has no known link to Kit and has the advantage over Kit that it does not label mast cells. Therefore we used DOG1 to study the distribution and fate of ICC [26].

The entire colon from the ileocecal junction to the pelvic brim was removed carefully, placed into the PBS (4°C), and the portions aboral to the occlusion clip, beneath the lip and oral to the clip separated from each other. For frozen sections, each segment was placed into optimal cutting temperature compound (OCT), quickly frozen with liquid nitrogen, and longitudinal sections (5 μm thickness) cut with a cryostat (Thermo Fisher Scientific, USA) and fixed with 100% acetone for 20 min (4°C). To obtain whole-mount preparations, each segment was inflated adequately with acetone overnight (4°C), and the longitudinal smooth muscle layer containing the ICC network at the level of the myenteric plexus was prepared with the aid of a dissection microscope.

The immunostaining procedures have been described previously [26]. Briefly, whole-mount preparations or frozen sections were placed in PBS containing 0.5% Triton X-100, 1% bovine serum albumin, and 10% normal goat serum for blocking for 1 h at room temperature to avoid nonspecific staining. Following blocking, whole mount preparations or frozen sections were incubated with DOG1 (1:100, Abcam, USA) overnight at 4°C. Alexa Flour 568-conjugated donkey anti-rabbit IgG (1:200, Invitrogen, USA) was used as secondary antibody. Preparations were counterstained with DAPI. Negative controls were performed by omission of primary antibody. The staining results were detected using a BX81 fluorescence microscope (OLYMPUS, Japan) or confocal laser scanning microscope (FV1000, OLYMPUS, Japan) with excitation wavelengths appropriate for Alexa Flour 568 (559 nm) and DAPI (405 nm). Photomicrographs of positive cells were taken in 10 random fields (×600 magnification, 0.2607 mm^2^) per whole-mount preparation.

### Measurement of inflammatory mediators

After measurement of intestinal myoelectric slow wave activity, a blood sample was collected from each animal and centrifuged at 2000 g for 15 min. Plasma was stored at −80˚C for subsequent measurements. Serum levels of TNF-α (ng/mL), IL-10 (pg/mL) and NO (IU/mL) were measured by immunoenzymatic enzyme-linked immunosorbent assay (ELISA, Quantikine High Sensitivity Rat by R&D Systems, USA) according to the manufacturer’s protocol. Optical density (OD) was detected using a spectrophotometer (Bio-Tek ELX800, USA).

### Western blot analysis

To confirm the changes in expression levels of c-Kit in each group, the segment of intestine oral to the occlusion clip was used for Western blot analysis. Total protein was extracted from the smooth muscle layers of the colon using RIPA lysis buffer (50 mM Tris pH = 7.4, 150 mM NaCl, 1% Triton X-100, 1% sodium deoxycholate, 0.1%SDS, 1 mM EDTA, 1 mM sodium orthovanadate, 10 mM NaF), containing 10 mg/mL aprotinin, 10 mg/mL leupeptin and 1 mM phenylmethylsulphonyl fluoride (PMSF) and 1 mM dithiothreitol (DTT) and centrifuged at 12000 g for 5 min. Protein concentration was then measured using a BCA Protein Assay Kit with bovine serum albumin as the standard (Beyotime Biotechnology, Jiangsu, China). Total protein (100 mg) was separated on 8% SDS-PAGE and transferred to a PVDF membrane (0.2 μm) at 300 mA for 2 h by using a wet transfer instrument (Bio-Rad, Hercules, CA, USA). To reduce background staining, the membrane was incubated with 5% non-fat dry milk in TBS containing 0.5% Tween 20 for 1 hour at room temperature, followed by incubation with c-Kit antibody (dilution, 1: 400; Santa Cruz Biotechnology, Santa Cruz, CA, U.S.A.) overnight at 4°C, then incubated with secondary anti-rabbit antibody conjugated with horseradish peroxidase for one hour at 37°C. Protein-antibody complexes were detected with an ECL Western blotting detection kit (Millipore, Billerica, USA). The levels of the measured protein were normalized to the level of GAPDH (KangChen Bio-tech, Shanghai, China). The result of the Western blot analysis was scanned, and the quantification of the Western blotting was done using Quantity One imaging software (Bio-Rad, Hercules, CA, USA).

### Statistical analysis

Results for continuous variables were given as means and standard deviations. One-way ANOVA was used to compare overall effects among groups. If the overall effect showed significant differences among groups, Bonferoni tests were used for two by two comparisons. The p values represent the results of the two by two comparisons from the Bonferoni post hoc tests. Statistical analyses used SPSS software version 17 (SPSS Inc, Chicago, IL, US). A two-tailed *p* of < 0.05 was considered significant.

## Results

### Animal model of partial bowel obstruction

**Figure 1:**
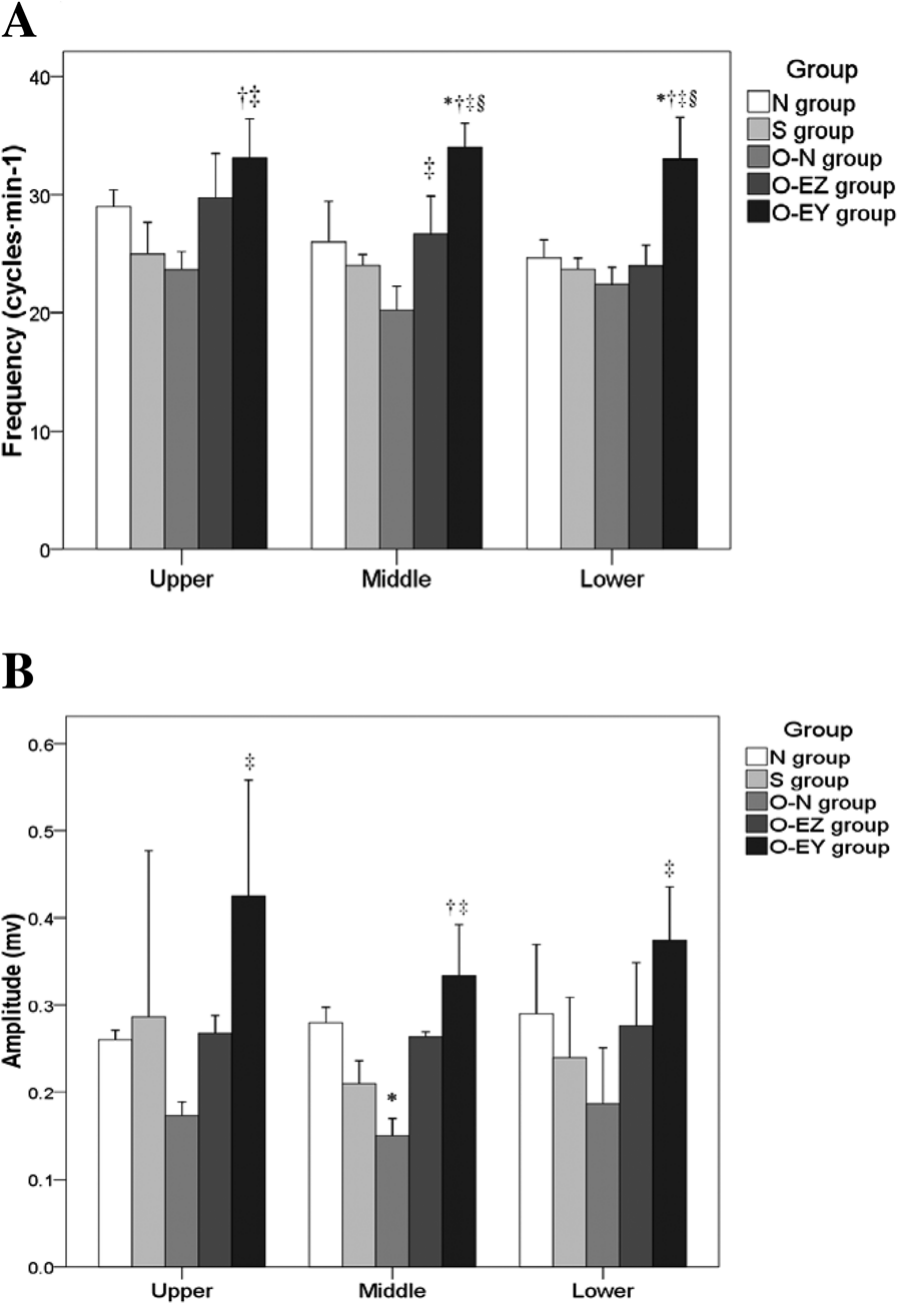
**Effect of electroacupuncture treatment on slow wave myoelectric activity of various intestinal segments in rats. (A)** Frequency (cycles · min^−1^) **(B)** Amplitude (mv). N group: Normal group; S group: Sham-operated group; O-N group: operated without EA treatment group; O-EZ group: operated and EA treatment at ST36 Zusanli acupoint group; O-EY group: operated and EA treatment at SP9 Yinglingquan acupoint group. *represents significant difference compared to N group. ^†^represents significant difference compared to S group. ^‡^represents significant difference compared to O-N group. ^§^represents significant difference compared to O-EZ group.

**Figure 2:**
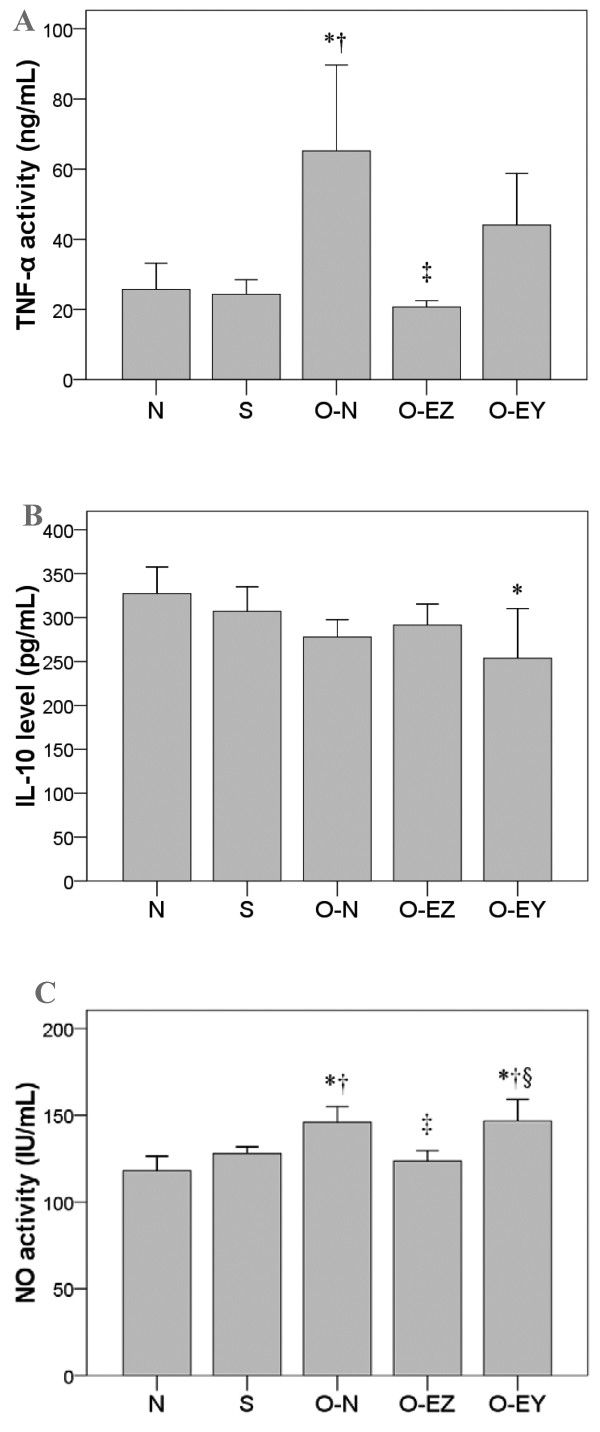
**Effect of EA on the serum expression of inflammatory mediators among groups. (A)** TNF-α activity (ng/mL) **(B)** IL-10 level (pg/mL) **(C)** NO activity (IU/mL) N group: Normal group; S group: Sham-operated group; O-N group: operated without EA treatment group; O-EZ group: operated and EA treatment at ST36 Zusanli point group; O-EY group: operated and EA treatment at SP9 Yinglingquan point group. *represents significant difference from N group. ^†^represents significant difference from S group. ^‡^represents significant difference from O-N group. ^§^represents significant difference from O-EZ group.

The animal model of partial bowel obstruction used in this study was obtained by placing a latex clip that partly occluded the bowel lumen over a section of the small intestine. When, in preliminary experiments, the intestine was examined fifteen days after the placement of the clip, significant distention (150-300%) was seen oral to the site of the clip in all animals (data not shown). During the course of the study, most of the clip-installed animals showed poorer food intake, constipation, and even difficult defecation. These observations confirmed the success of the model.In addition to the distension and hypertrophy seen oral to the clip, animals with partial obstruction (but no EA treatment) had decreased slow wave amplitude and frequency in all 3 bowel segments (Figure 1), increased serum levels of TNF-α and NO (Figure 2), and decreased levels of c-Kit, results indicating that partial obstruction led to a loss of normal contractile control, an increase in inflammatory activity, and decreased numbers of ICCs.

#### Effects of electroacupuncture treatment on slow wave myoelectric activity of various intestinal segments

Figure 1 shows the effect of EA on myoelectric activity in the partially obstructed bowel. Partial bowel obstruction itself caused a decrease in the frequency and amplitude of slow waves oral to the obstruction, at the obstruction, and aboral to the obstruction, but this decrease only reached statistical significance for amplitude at the site of the obstruction. EA at the control acupoint, SP9, significantly increased frequency and amplitude of the slow waves compared to those in the untreated obstruction. EA at the test acupoint, Zusanli, showed a consistent trend for increased slow wave frequency and amplitude, but this trend only reached significance for frequency at the site of the obstruction.

Comparisons with non-EA groups of the effects of EA at upper, middle, and lower bowel segments are shown in Figure 1A and 1B. Slow wave frequencies were significantly higher in O-EY group compared with S and O-N groups at upper segment (33.13 vs. 25.00, 23.67 cycles · min^−1^; p = 0.007, 0.002), and were significantly higher compared to other four groups at middle and lower segments (middle: 34.06 vs. 26.00, 24.01, 20.23, 26.67 cycles · min^−1^; p = 0.001, <0.001, <0.001, 0.003; lower: 33.02 vs. 24.67, 23.68, 22.39, 24.00 cycles · min^−1^; p = 0.003, 0.001, <0.001, 0.001). The frequency in the O-EZ group at the middle part was significantly higher than in the O-N group (26.67 vs. 20.23 cycles · min^−1^, p = 0.040) (Figure 1A).Significantly higher amplitudes in the O-EY group compared with the O-N group were observed at all three parts (upper: 0.43 vs. 0.17 mv, p = 0.031; middle: 0.33 vs. 0.15, p < 0.001; lower: 0.38 vs. 0.19 mv, p = 0.008). In addition, the amplitude in the middle part was significantly lower in the O-N group compared with the N group (0.15 vs. 0.28 mv, p = 0.019); and was significantly higher in the O-EY group than the S group (0.33 vs. 0.21, p = 0.006) (Figure 1B).

#### Effects of EA on the expression of inflammatory mediators

Partial bowel obstruction significantly increased serum levels of the 2 inflammatory mediators, TNF-α and NO, but had no significant effect on levels of the anti-inflammatory cytokine, IL-10. EA at the Zusanli acupoint significantly reduced the partial obstruction-related increase in TNF-α and NO. In contrast, EA at the control acupoint had no effect.The comparisons in serum levels of TNF-α, IL-10, and NO among groups are shown in Figure 2A, 2B, 2C. Serum levels of TNF-α were significantly higher in the O-N group compared to the N, S and O-EZ groups (65.19 vs. 25.69, 24.31, 20.69 ng/mL; p = 0.004, 0.003, <0.001) (Figure 2A).The serums level of IL-10 was lower in the O-EY group than the N group (253.70 vs. 327.41 pg/mL, p = 0.028) (Figure 2B). The serum level of NO activity was significantly higher in the O-N and O-EY groups than in the N and S (145.97 and 146.72 vs. 117.96 and 127.83 IU/mL; p ≤ 0.027) groups. NO activity levels were lower in the O-EZ group compared with the O-N and O-EY groups (123.49 vs. 145.97 and 146.72 IU/mL, p = 0.004 and 0.003). No significant differences were found in the N or S groups compared to the O-EZ group (117.96, 127.83 vs. 123.49 IU/mL, p > 0.05) (Figure 2C).

#### Effects of EA on c-Kit levels

**Figure 3:**
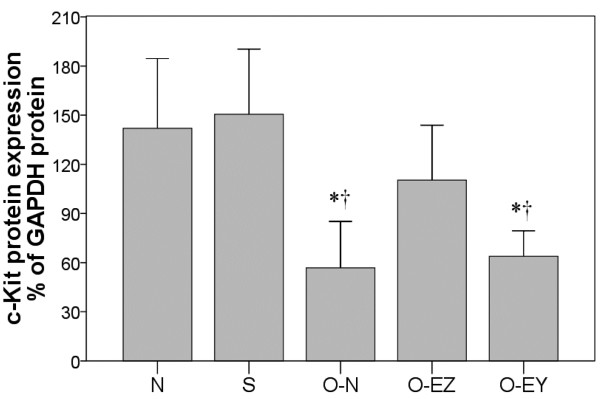
**Effects of EA on c-Kit levels among groups.** N group: Normal group; S group: Sham-operated group; O-N group: operated without EA treatment group; O-EZ group: operated and EA treatment at ST36 Zusanli point group; O-EY group: operated and EA treatment at SP9 Yinglingquan point group. *represents significant difference from N group, ^†^represents significant difference from S group. ^‡^represents significant difference from O-N group. ^§^represents significant difference from O-EZ group.

Partial bowel obstruction significantly lowered levels of c-Kit protein, the marker for ICC, in intestinal tissue. EA at Zusanli increased this lower level of c-Kit protein to levels not significantly different from those seen in normal and sham-operated rats. EA at the control acupoint had no effect on c-Kit protein levels.Comparisons between groups in c-Kit protein expression are shown in Figure 3. The c-Kit protein expression was significantly lower in the O-N and O-EY groups compared to the N and S groups (56.84 and 63.88 vs. 141.99, 150.55%; p ≤ 0.014).

#### Effects of EA on distribution and alteration of ICCs

**Figure 4:**
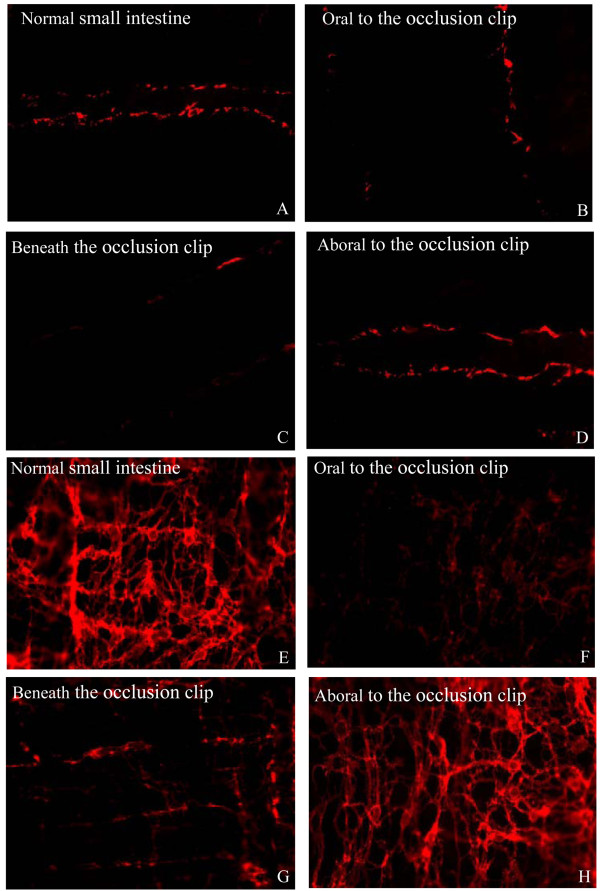
**Confocal images showing the ICCs in the rat colon immunolabeled for DOG1. (A-D)** on frozen sections, **(E-H)** on whole-mount preparations, **(A, E)**: normal small intestine, **(B, F)**: oral to the occlusion clip, **(C, G)**: beneath the occlusion clip, **(D, H)**: aboral to the occlusion clip. In the normal small intestine, DOG1-like immunoreactivity (DOG1-LI) was localized in two distinct populations of ICC (ICC-MY and ICC-DMP) within the tunica muscularis and DOG1-LI ICCs were normally present with famose processes and intact cellular networks **(A, E)**. Beneath and oral to the clip, the density of DOG1-LI ICCs were significantly reduced, the distribution was not continuous, and ICC networks were partly damaged **(B, C, F, G)**. Aboral to the clip, ICC networks and function were essentially normal **(D, H)**.

**Figure 5:**
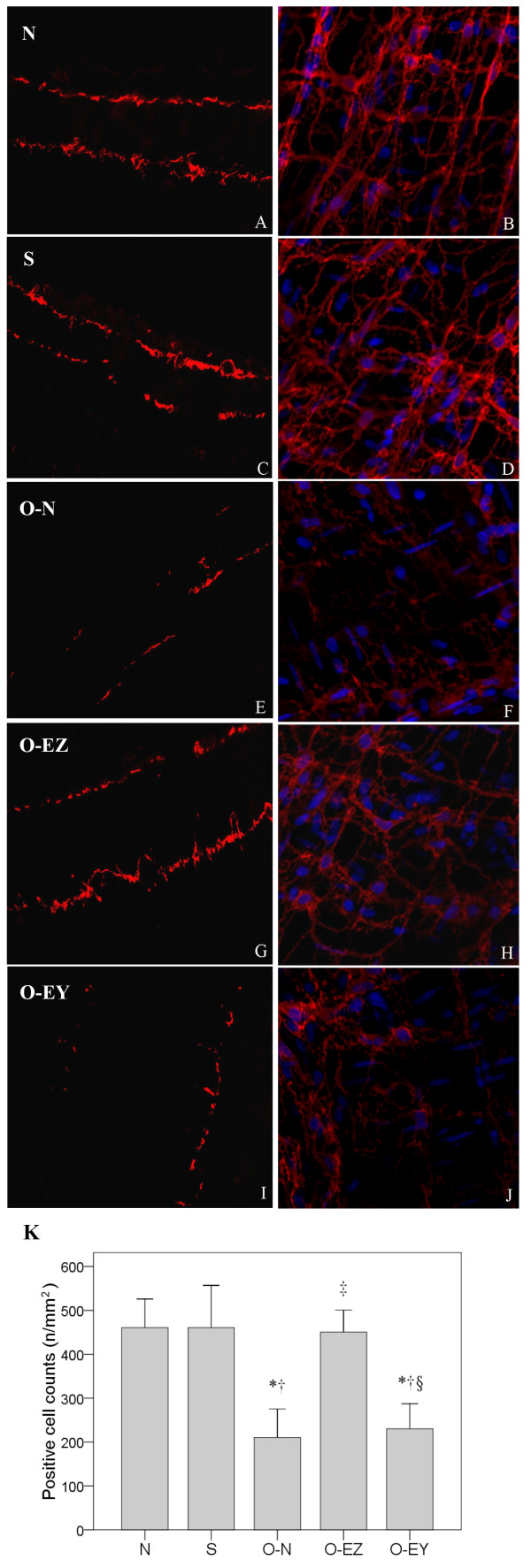
**Confocal images of ICC oral to the clip labeled with DOG1 on frozen sections (A, C, E, G, I) and on whole-mount preparations (B, D, F, H, J) showing the alterations of distribution and morphology in control (A, B), sham-operated (C, D), operated without EA treatment group (E, F), operated and EA at Zusanli (G, H), operated and EA treatment at Yinglingquan (I, J).** In the N and S group, DOG1-like immunoreactivity (DOG1-LI) was localized in two distinct populations of ICC (ICC-MY and ICC-DMP) within the tunica muscularis and DOG1-LI ICCs were normally present with famose processes and intact cellular networks **(A-D)**. In the O-N group, the density of DOG1-LI ICCs were significantly reduced, the distribution was not continuous, and ICC networks were partly damaged **(E, F)**. In the O-EZ group, an almost intact cellular networks could be observed and DOG1+ cell numbers had recovered to control values **(G, H)**. In the O-EY group, the DOG1^+^ cell density was still with the same to the O-N group **(I, J)**. Comparison between groups in positive cell counts **(K)**. N group: Normal group; S group: Sham-operated group; O-N group: operated without EA treatment group; O-EZ group: operated and EA treatment at ST36 Zusanli point group; O-EY group: operated and EA treatment at SP9 Yinglingquan point group. *represents significant difference from N group, ^†^represents significant difference from S group. ^‡^represents significant difference from O-N group. ^§^represents significant difference from O-EZ group.

DOG1 is a Kit-independent marker for ICC that is reported to have superior sensitivity and specificity compared to Kit (CD117) and CD34 [6-8]. Therefore, we used DOG1 as the marker to examine the distribution and alteration of ICCs in our study (Figure 4). Our results showed that, in the N and S groups, DOG1-like immunoreactivity (DOG1-LI) was localized in two distinct populations of ICC, one population with processes and numerous branches forming a cellular network between the circular and longitudinal muscle layers at the level of the myenteric plexus (ICC-MY), and a second population at the level of the deep muscular plexus in the circular muscle layer (ICC-DMP). In the O-N group, we found that these ICC networks showed a gradient in which severity of damage decreased from oral to aboral. Oral to the clip, on frozen sections the density of DOG1^+^ ICC was significantly reduced and the distribution was not continuous compared to the control group (Figure 4B), and on the whole-mount preparations, ICC networks were partly damaged, and the numbers of DOG1 and DAPI double-labeled cells were also significantly reduced (Figure 4F), indicating that some ICCs might be absent. Aboral to the obstruction, ICC networks were essentially normal (Figure 4D, 4H). Figure 5 shows the results of EA with Zusanli and SP9 on the oral segment. In the O-EZ group, an almost intact cellular network could be observed and DOG1^+^ cell numbers had recovered to control values (Figure 5G&H). In the O-EY group, the DOG1^+^ cell density was still with the same as in the O-N group (Figure 5 I&J). The result indicated that EA at Zusanli may stimulate the recovery of ICCs in partial bowel obstruction in rats.

Comparisons between groups in positive cell counts per mm^2^ are shown in Figure 5K. The positive cell counts were significantly lower in O-N and O-EY groups compared with N, S and O-EZ groups (210.33 and 230.36 n/mm^2^ vs. 460.71, 460.71 and 450.70 n/mm^2^, p < 0.001).

## Discussion

In our rat model, partial bowel obstruction caused an increase in inflammatory mediators, no change in the anti-inflammatory mediator IL-10, and decreases in slow wave activity and ICC number, results agreeing with the results of others on the involvement of inflammation, ICC number, and myoelectric activity in bowel disorders. EA at the Zusanli, the test acupoint, increased the plasticity of ICC, that is, the return of transdifferentiated, inactive ICC to their original c-Kit positive phenotype. And this action may contribute to its reported usefulness in treatment of bowel disorders. But our results also revealed a paradox.

EA at Zusanli blocked the increase in inflammatory mediators and decrease in ICC number that occurred after partial bowel obstruction in our rat model of this condition. But EA at the Zusanli acupoint in general showed only a trend to increase myoelectric activity, although it did show a significant increase in slow wave frequency at one location, the location of the obstruction. EA at the control acupoint, in contrast, very significantly increased myoelectric activity in all three segments of the intestine, but had no effect on inflammatory mediators or ICC number.

A question posed by these results is how EA at SP9, the control acupoint, could increase myoelectric activity without correcting the ICC depletion caused by partial bowel obstruction. Functioning ICC are essential for slow wave activity in the GI tract [30]. These cells have a set of ion channels that enable them to undergo spontaneous rhythmic depolarizations and repolarizations, are connected to each other and to smooth muscle cells by gap junctions, and are therefore able to provide pacemaker activity to the GI smooth muscle [9,17,31]. Smooth muscle cells and enteric neurons do not have this pacemaker ability, and if ICC are not present or their function is blocked, slow waves cannot be generated. However ICC are also in contact with enteric neurons [31], and vagal afferents contact both the ICC and GI smooth muscle [32]. The autonomic nervous system (ANS) is able through sympathetic or parasympathetic output to modulate the resting potential and ion currents of the ICC to either increase or decrease the ICC-generated slow waves and thus influence gut motility. The ANS can also affect smooth muscle contractility directly. Acupoints are locations on the skin of low electrical resistance that are thought to provide special pathways for the conduction of electrical signals. EA at the Zusanli acupoint has been shown to increase afferent input into the dorsal motor nucleus of the brain stem in freely moving rats and increase parasympathetic output [23]. EA at this acupoint has also been shown to affect the subgranular zone of the dentate gyrus [33,34] and the medial tractus solitarius [25]. We have no data on whether EA at SP9, the control acupoint, affects ANS control of GI motility. But from the results seen in the current study, one might hypothesize that some ICC remain in the oral portion of the partially obstructed bowel, and that EA at SP9 increases myoelectric activity by causing a strong vagal stimulation of the remaining ICC and the smooth muscle.

The pathology seen in our partial obstruction model was similar to that reported by others – hypertrophy above the obstruction, decreased slow wave amplitude and frequency, and decreased ICC oral to the obstruction [17,25]. ICC restoration without the use of EA has been reported in a number of situations - - after intestinal transection and anastomosis [35], when blockade of c-Kit is removed [14], and in the partially obstructed small intestine [36], But no previous studies of the effect of EA on ICC number or function in partial bowel obstruction have been reported.

Our results show EA at Zusanli to increase ICC number, but to do so in a manner not closely related to myoelectric activity. Therefore it may act through some other mechanism in increasing ICC. Previous studies have implicated local inflammatory activity as a possible cause of decrease in ICC numbers. Resident macrophages are the most likely source of this inflammatory activity [17]. In a previous study of a rat model of partial bowel obstruction, macrophage number was reported to increase on the oral side of the bowel obstruction although no leukocyte infiltration or other signs of inflammation were seen [20]. At the same time in this preparation, TNF-α levels increased, spontaneous contractions decreased, and there was a reduction in ICC number oral to the occlusion. Macrophage numbers have also been reported to increase in an animal model of Hirschsprung’s disease [37], a condition that also involves partial bowel obstruction. Increased numbers of local macrophages are likely, therefore, to be the source of the increased TNF-α seen in our experiment.

The association in our results of increased serum TNF-α with decreased ICC numbers and decreased TNF-α with increased ICC numbers suggests that TNF-α increase might be a cause of the decrease in ICC number. For ICC to function, the tyrosine kinase receptor c-Kit must be present on the cell surface and be activated by binding to cell-bound SCF. The neighboring intestinal smooth muscle cells have the necessary bound SCF to activate c-Kit and maintain ICC function. The effect of TNF-α on the SCF/c-Kit interaction, therefore, needs to be more fully investigated.

EA at the control acupoint in our experiment greatly increased the magnitude and frequency of the slow waves, but had no effect of TNF-α and ICC number. This shows that there is no tight reciprocal cause and effect relationship between slow wave strength and ICC number. It also indicates that the effect of EA at Zusanli must be through another mechanism than the mechanism influencing slow wave activity. Studies of the areas of the brain receiving afferent input from Zusanli acupuncture show a variety of locations receiving this input [38,39]. The possibility that EA at Zusanli, acting at a different CNS location from SP9 EA, causes release of an endocrine factor or neuromediator that affects macrophages and TNF-α release also needs investigation.

## Conclusion

In conclusion, we have demonstrated that EA at Zusanli can restore ICC networks of the partially obstructed small intestine in rats and decrease release of the inflammatory mediator, TNF-α. However, myoelectric activity could be greatly increased by stimulation at another acupoint, SP9, without any accompanying increase in ICC or decrease in TNF-α. Therefore more research is needed on the relationship between EA, ICC, and myoelectric activity in order to determine the potential usefulness of this therapy for STC patients with GI motility disorders.

## Competing interests

The authors declare that they have no competing interests.

## Authors’ contributions

LZ put forward the study concepts and guaranteed the integrity of the entire study. KL, LJT and LZ designed the study and defined the intellectual content. MFP, KL, LJT, and LZ performed the literature research. CW, XYZ, ZY, GHZ, PHW, and YHW acquired the data. MFP, KL, LJT, LZ analyzed the data and statistics. MFP, KL, LJT, LZ prepared and edited the manuscript. CW, XYZ, ZY, GHZ, PHW, and YHW revised the manuscript. All authors read and approved the final manuscript.

## Pre-publication history

The pre-publication history for this paper can be accessed here:

http://www.biomedcentral.com/1472-6882/14/186/prepub
